# Caregiver Stress Is Improved and Sustained Following Transcatheter Aortic Valve Replacement

**DOI:** 10.1089/pmr.2022.0064

**Published:** 2023-02-24

**Authors:** Mohammed Elzeneini, Alfaroug Osman, Khadija Bright, Shuang Yang, Yi Guo, Eric Jeng, Khanjan B. Shah

**Affiliations:** ^1^ Division of Cardiovascular Medicine, University of Florida, Gainesville, Florida, USA.; ^2^ Department of Health Outcomes and Biomedical Informatics, University of Florida, Gainesville, Florida, USA.; ^3^ Division of Thoracic and Cardiovascular Surgery, University of Florida, Gainesville, Florida, USA.

**Keywords:** aortic stenosis, caregiver issues, palliative care

## Abstract

**Background:**

Little is known about the impact of symptomatic aortic stenosis and subsequent transcatheter aortic valve replacement (TAVR) on stress and health for the caregiver. In this prospective cohort study, we measured caregiver stress before and after TAVR.

**Methods:**

We interviewed 34 primary caregivers for patients undergoing outpatient TAVR at an academic institution. Caregiver stress was measured using the Kingston Caregiver Stress Scale (KCSS) and the Caregiver Self-Assessment Questionnaire (CSAQ) before TAVR and at one and six months after. Mean scores were compared pre- and post-TAVR using the Wilcoxon signed-rank test.

**Results:**

There was significant improvement in KCSS caregiver stress at one month that was sustained at six months post-TAVR (mean change −1.91 ± 2.50 for six months, *p*-value 0.01). This was primarily driven by improvement in caregiving issues rather than family or financial issues. There was also significant improvement in CSAQ self-assessed health/illness at one and six months (mean change −2.78 ± 4.01 for six months, *p*-value 0.016).

**Conclusions:**

Our findings support further investigation of caregiver outcomes in shared decision making before TAVR.

## Background

Patients with severe symptomatic aortic stenosis are frequently limited by symptoms of angina and heart failure and are at risk for recurrent admissions.^1^ The prevalence of clinically significant or severe aortic stenosis in the elderly U.S. population is about 3.5%,^2,3^ with 85% of them requiring hospitalization.^2^ Caregiver stress has been associated with negative health outcomes, such as depression and increased mortality.^4^ The impact of severe symptomatic aortic stenosis and subsequent transcatheter aortic valve replacement (TAVR) on caregiver stress has not been studied in a contemporary U.S. population. Owing to the high likelihood that TAVR relieves symptoms of heart failure, we hypothesize that caregiver stress improves after TAVR. In this pilot study, we seek to measure the burden of caregiver stress in patients with severe aortic stenosis and the change in caregiver stress after TAVR.

## Methods

We interviewed the primary caregiver for 34 consecutive patients undergoing elective outpatient TAVR for severe symptomatic aortic stenosis at the University of Florida between January and November 2021. Caregiver stress was measured using two previously validated questionnaires: the Kingston Caregiver Stress Scale (KCSS; 10–50, lowest–highest stress)^5^ and the Caregiver Self-Assessment Questionnaire (CSAQ).^6^ These tools were validated in caregivers of patients with dementia.^5,6^ The KCSS consists of 10 questions grouped into three categories: caregiving, family, and financial issues.

The CSAQ consists of 18 items, with the final 2 items assessing self-perceived health and stress on a scale of 1 to 10 (10 being highest stress/illness). The KCSS and CSAQ were administered by a trained interviewer to the caregiver at outpatient clinic visits before TAVR and at one and six months post-TAVR. Mean scores at baseline, one, and six months were compared using the nonparametric Wilcoxon signed-rank test. A *p*-value <0.05 for change in mean scores was considered statistically significant. This study was approved by the University of Florida Institutional Review Board.

## Results

Thirty-four caregivers completed the pre-TAVR questionnaires, 31 completed the one-month and 18 completed the six-month follow-up questionnaires. Eight caregivers were lost to follow-up, and three and five caregivers declined to participate at one and six months, respectively. The mean pre-TAVR caregiver stress as measured by KCSS was 14.60 out of 50 (standard deviation [SD] 5.32), whereas the mean CSAQ self-assessed health/illness was 4.80 out of 10 (SD 3.60).

There was significant improvement in caregiver stress as measured by KCSS at one month post-TAVR (mean change −1.21 ± 2.59, *p*-value 0.02), and this improvement was sustained at six months post-TAVR (mean change −1.91 ± 2.50, *p*-value 0.01) (Table 1). The reduction in KCSS score was primarily driven by improvement in caregiving issues rather than family or financial issues as seen in Table 1. There was also significant improvement in CSAQ self-assessed health/illness at one month post-TAVR (mean change −1.93 ± 3.04, *p*-value <0.001), which was sustained at six months post-TAVR (mean change −2.78 ± 4.01, *p*-value 0.016). There was no change in other items of the CSAQ.

**Table 1.** Difference in Kingston Caregiver Stress Scale Caregiver Stress and Caregiver Self-Assessment Questionnaire Self-Assessed Stress and Health/Illness Pre-Transcatheter Aortic Valve Replacement and at One and Six Months Post-Transcatheter Aortic Valve Replacement

	Pre-TAVR, mean (SD)	Post-TAVR, mean (SD)	Change, mean (SD)	*p*-Value^a^
At one month post-TAVR (*n* = 31)				
KCSS total stress Q1-10: score 10–50	14.60 (5.32)	12.79 (5.05)	−1.21 (2.59)	0.021
KCSS caregiving issues Q1-7: score 7–35	10.45 (4.48)	9.27 (3.99)	−1.18 (2.60)	0.022
KCSS family issues Q8-9: score 2–10	2.29 (0.99)	2.16 (0.73)	−0.13 (0.63)	0.500
KCSS financial issues Q10: score 1–5	1.38 (0.65)	1.45 (1.15)	0.07 (0.58)	1.000
CSAQ self-assessed stress Q17: score 1–10	3.19 (2.3)	2.55 (2.62)	−0.64 (2.24)	0.111
CSAQ self-assessed illness Q18: score 1–10	4.80 (3.60)	2.87 (2.29)	−1.93 (3.04)	<0.001
At six months post-TAVR (*n* = 18)				
KCSS total stress Q1-10: score 10–50	14.63 (5.32)	12.72 (6.36)	−1.91 (2.50)	0.011
KCSS caregiving issues Q1-7: score 7–35	10.89 (4.40)	9.50 (3.82)	−1.39 (2.54)	0.037
KCSS family issues Q8-9: score 2–10	2.23 (0.73)	2.06 (0.23)	−0.17 (0.51)	0.500
KCSS financial issues Q10: score 1–5	1.23 (0.73)	1.17 (0.51)	−0.06 (0.23)	1.000
CSAQ self-assessed stress Q17: score 1–10	2.72 (1.60)	2.78 (1.86)	−0.06 (2.15)	0.799
CSAQ self-assessed illness Q18: score 1–10	5.00 (3.69)	2.22 (1.35)	−2.78 (4.01)	0.016

^a^ Based on Wilcoxon signed-rank test.

CSAQ, Caregiver Self-Assessment Questionnaire; KCSS, Kingston Caregiver Stress Scale; SD, standard deviation; TAVR, transcatheter aortic valve replacement.

## Discussion

Caregiver stress is a novel patient-centered outcome that has been associated with adverse outcomes in other disease states.^4^ In cardiovascular disease, caregiver stress has been associated with poor quality of life,^7^ calling for efforts to develop clinical interventions to support caregivers and improve their quality of life.^8^ The impact of severe aortic stenosis and subsequent TAVR on caregiver stress has not been previously studied in a contemporary U.S. cohort. In this prospective study, we demonstrate that overall caregiver stress of the cohort before TAVR is relatively low, suggesting that symptomatic aortic stenosis is associated with less caregiver stress compared with conditions such as cancer.^9^ Caregiver self-assessed health/illness before TAVR is also below what would require seeking medical advice (≥6).^6^

Our data suggest that treatment of severe symptomatic aortic stenosis with TAVR was associated with an improvement in caregiver stress at one month that was sustained at six months. Caregivers also perceived an improvement in their overall health. Shared decision making with patients and families is a cornerstone of multidisciplinary decision making before TAVR, and our findings suggest further investigation of caregiver stress in this space. Areas of future investigation include incorporating caregiver outcomes prospectively in TAVR trials with larger sample sizes and using qualitative methodology to fully understand features contributing to caregiver stress as it relates to structural heart disease. Other aspects of caregiver outcomes that would need to be studied include depressive symptoms, anxiety, and quality of life, all of which are associated with overall caregiver stress.^5^

## Abbreviations Used

CSAQ: Caregiver Self-Assessment Questionnaire
KCSS: Kingston Caregiver Stress Scale
SD: standard deviation
TAVR: transcatheter aortic valve replacement
